# Understanding Nevoid Basal Cell Carcinoma Syndrome (Gorlin Syndrome): A Case Report

**DOI:** 10.7759/cureus.36537

**Published:** 2023-03-22

**Authors:** Yisia Olivero, Jonathan Otero-Colón, Samin Rahman, Brandon Grodman, Vilma Vas

**Affiliations:** 1 Internal Medicine, Nassau University Medical Center, East Meadow, USA; 2 Internal Medicine, American University of the Caribbean, East Meadow, USA

**Keywords:** odontogenic keratocyst, ocular anomalies, nbccs, nevoid basal cell carcinoma, gorlin syndrome

## Abstract

To date, there is no definite effective target therapy or cure for nevoid basal cell carcinoma syndrome (NBCCS, Gorlin syndrome). Basal cell carcinoma is frequently the far most increased risk of this syndrome, including predisposition to other malignancies. In 2015, an 11-year-old female with a past medical history of sickle cell trait, oral, and unilateral knee abscesses presented with multiple visits for various nodules covering the hands and chest, as well as posterior knee cysts. Genetic testing confirmed the diagnosis. The key to treatment and surveillance relies on appropriate recognition, management of atypical presentations, and offering appropriate genetic counseling to families.

## Introduction

Nevoid basal cell carcinoma syndrome (NBCCS), also known as Gorlin syndrome, is a rare genetic disease. NBCCS is an autosomal dominant disorder characterized by developmental defects and tumorigenesis [1]. The gene responsible for NBCCS is PTCH1, which encodes a receptor for the secreted protein, sonic hedgehog [1]. Clinically, there have been numerous abnormalities reported in the NBCCS. In 1993, Evans et al. reviewed 84 cases, looking at the common complications observed in NBCCS along with clearer guidelines for screening and counseling [2]. The first step of evaluation is to appropriately achieve a diagnosis. The diagnosis of NBCCS can be established using one major criterion and genetic confirmation, two major criteria, or one major criterion with two minor criteria [1]. The major criteria consist of one basal cell carcinoma (BCC) under the age of 20 years or multiple BCCs; an odontogenic keratocyst of the jaw proven by histology in an individual younger than 20 years; two or more palmar or plantar pits; lamellar (sheet-like) calcification of the falx cerebri or clear evidence of calcification in an individual younger than the age of 20 years; childhood medulloblastoma; a first-degree relative with NBCC [2]. Whereas minor criteria consist of rib anomalies (bifid, fused, or markedly splayed ribs); macrocephaly; cleft lip or palate; other specific skeletal abnormalities (vertebral kyphoscoliosis, short fourth metacarpals, postaxial polydactyly); lymphomesenteric cysts; ovarian or cardiac fibroma; ocular anomalies (strabismus, hypertelorism, congenital cataracts, glaucoma, coloboma) [2]. NBCCS is sometimes diagnosed in very young patients, but in most cases, it occurs in people aged between 17 and 35 years [2]. The condition is very difficult to diagnose in early childhood because its symptoms appear gradually as the child develops [2]. After a diagnosis is made, management of NBCSS requires a multidisciplinary approach, including surveillance for the development of syndrome-related complications such as basal cell carcinomas and, to a lesser extent, medulloblastomas [2]. Surveillance in individuals with NBCCS may be varied based on genetic variance due to more relevant complications. Our patient presented a unique constellation of abnormalities that required an elevated level of suspicion for diagnosis.

## Case presentation

**Figure 1:**
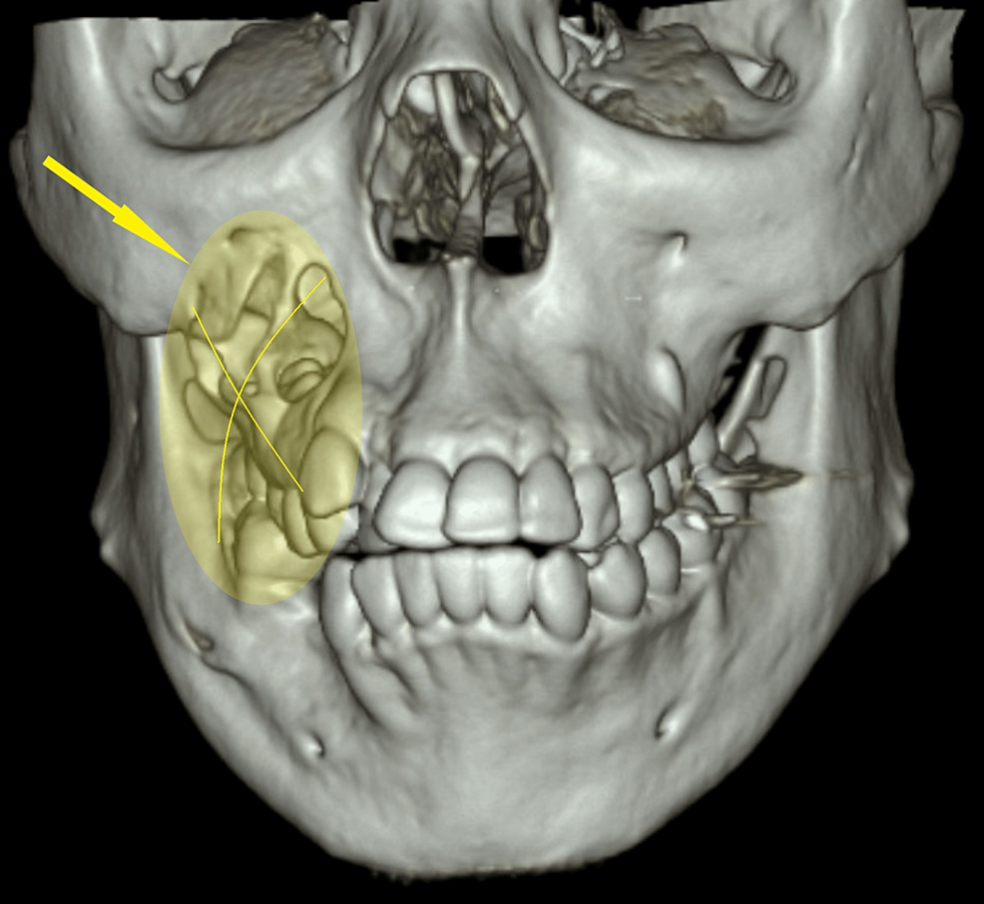
CT maxillofacial w/o contrast: 3D view 3.4 cm × 3.4 cm dentigerous cyst of the right maxillary sinus with expansion thinning of the surrounding maxillary sinus walls.

**Figure 2:**
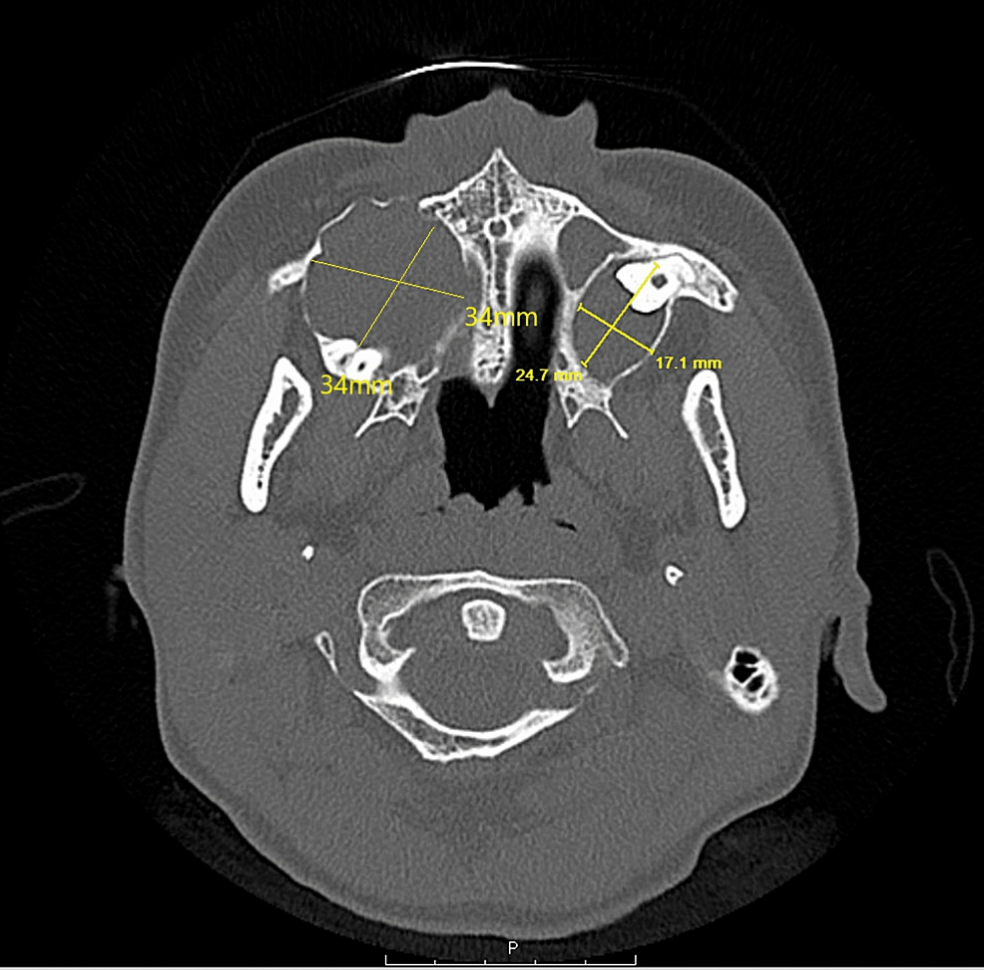
CT maxillofacial w/o contrast There is a 1.7 cm × 2.4 cm left-sided dentigerous cyst with expansion of the posterior maxillary wall and 3.4 cm × 3.4 cm dentigerous cyst of the right maxillary sinus with expansion thinning of the surrounding maxillary sinus walls.

An 11-year-old female presented to primary care with a significant past medical history of sickle cell trait, oral and unilateral knee abscesses in 2009, and previous mandibular surgery. The patient presented with multiple visits for multiple nodules covering the hands, chest, and posterior knee cysts measuring 2 cm each. Notable physical characteristics include exophthalmos, a flat midface, multiple scattered palmar pits, subcutaneous venous markings, and scoliosis. These findings met major criteria for high suspicion for NBCCS despite the early onset of age. In December 2015, genetic testing confirmed the diagnosis. The patient was lost to follow-up, and in 2021, she developed recurring cysts with complications including a ruptured ovarian cyst, a maxillary sinus cyst causing proptosis, a dentigerous cyst of the right maxillary sinus with expansion thinning of the surrounding sinus walls (Figure 1), and a left-sided cyst with expansion into the posterior maxillary wall (Figure 2). These complications prompted a return to the emergency department for treatment and the re-establishment of primary care. The ophthalmologic evaluation demonstrated proptosis in the right eye secondary to maxillary sinus cysts encroaching on the orbit.

The patient underwent incisional biopsy and decompression of multiple lesions of the bilateral maxilla and mandible by oral and maxillofacial surgery (OMFS). Biopsies demonstrated odontogenic keratocysts, another major criterion for diagnosis. Status: post-surgical intervention, close monitoring is required to evaluate for recurrence and new cyst formation. Our patient requires periodic maintenance and surveillance from a multidisciplinary team consisting of primary care and OMFS as well as other specialties depending on the location and involvement of cyst formation.

## Discussion

The highest incidence rate of NBCCS is found between puberty and 35 years of age, with a small incidence seen in children aged three to four years old and the youngest ever reported at two years of age [3]. However, the diagnosis rate is highest in Caucasians greater than 40 years of age. This case highlights an atypical subgroup of the population diagnosed with Gorlin syndrome, displaying her first symptoms appearing prior to puberty around the age of seven to eight and being found in an African American. For comparison, we describe a typical case of Gorlin syndrome subsequently.

Another reported case of Gorlin syndrome discussed a 25-year-old patient with a chief complaint of swelling in her cheeks bilaterally. This contrasting case demonstrates a more common age of presentation while also bringing to light a clinical presentation that differs from that of the patient being presented. The contrasting patient presented with facial milia, macrocephaly, and frontal bossing, as seen in 60% of patients with NBCCS [4]. Sixty percent of patients with NBCCS have a recognizable appearance with macrocephaly, frontal bossing, coarse facial features, and facial milia [5,6]. Most patients have skeletal anomalies, including bifid ribs and wedge-shaped vertebrae [5]. Additionally, 70-80% of NBCCS individuals have affected parents, leaving 20-30% with de novo pathogenic variants [5].

After establishing a diagnosis through mutation identification, recommendations for the surveillance of patients may be followed. Using discussions from the AACR Childhood Cancer Predisposition Workshop in 2016, surveillance recommendations have been published [3]. The recommendations for PTCH1 mutation carriers are annual basal cell carcinoma screening at age 10, a baseline echocardiogram to be performed during infancy, as well as dental exams with a concurrent jaw X-ray every 12-18 months beginning in childhood. There is a relatively lower risk of medulloblastoma; however, changes in the gross neurological exam or changes in head circumference may warrant further clinical investigation. These changes warrant a brain MRI every four months up until age 3, then every six months up until age 5 [3]. In addition, for females, ovarian ultrasound is recommended by age 18 [3,7]. Radiation-sparing treatment is recommended due to the risk of radiation-induced skin cancers [3]. Surgically, each situation must be evaluated. In fact, based on the extent of the keratocysts, the histology, the location and characteristics of the patient, and the advantages and disadvantages related to each surgical approach, they must be evaluated [8]. As for the relapse rate associated with the use of one technique over another, the literature reports OKC is a typical developmental cyst with a recurrence rate of 12% to 58.3% [8]. This involves extensive and routine maintenance. The follow-up should involve performing an orthopantomography every six months in young patients and a CBCT in case of doubt or to evaluate contiguity with anatomical structures such as neurovascular bundles, teeth, and maxillary sinuses [8].

When examining this case, the patient encompasses a unique population of patients diagnosed with NBCCS. This patient’s first incidence of cysts is noted to have presented at a prepubescent age, correlating with a smaller incidence of Gorlin syndrome patients [3]. Additionally, this patient is in the minority demographically.

## Conclusions

This case highlights an atypical presentation of Gorlin syndrome at an age younger than is typically seen. It highlights the importance of a potentially broader clinical threshold for pursuing diagnostic confirmation of Gorlin syndrome so that the appropriate screenings and multidisciplinary approach to long-term management can be started at an earlier time. Management consists of a multidisciplinary approach to monitoring and evaluating cysts as they are diagnosed. As for the surgical treatment of cystic lesions typical of the pathology at the level of the jawbones, there is currently no treatment of choice for resolution. Monitoring consists of close follow-up and specific evaluation of any complaints with appropriate imaging. In this case, we explain the importance of a low threshold for testing patients who present a high suspicion for Gorlin syndrome despite a lower incidence as well as the necessity for monitoring and screening to avoid or manage occurrences of cysts and malignancy.
